# Dental Caries

**Published:** 1884-12

**Authors:** 


### Dental Caries.

### A CRITICAL SUMMARY, AND THE PREVENTION OF DENTAL CARIES.

A Series of Papers, Reprinted from the Journal of the British Dental Association, by HENRY SEWILL, M.R.C.S. and L.D.S., England.

The papers composing this work appeared in the journal of the British Dental Association, but that they might be in a connected form, they have been revised, and are published in this little volume, which gives them a permanent form. Dr. Sewill presents in this volume a very good resume of the leading facts, so far as they are known, and opinions of those who have studied this subject, together with his own views. The work is well worthy of attentive perusal and study. Caries of the teeth is one of the most important subjects to which the attention of the dentist can be directed, and everything that will aid in perfecting a knowledge of the subject is well worthy of special attention. Dr. Sewill criticises quite sharply the views of some other members of the profession who have given considerable attention to the subject. There may be a difference of opinion as to the propriety of this kind of criticism, but investigators sometimes place themselves in such false positions that only the sharp crack of the whip will arrest their attention, or cause them to look in any other direction than that to which they have become so especially attached. A close reading of this little volume will be not only interesting, but instructive, to every member of our profession, and those familiar with Dr. Sewill's writing will be especially pleased to be in possession of this little manual.

